# Pot/Lid Illusion

**DOI:** 10.1177/2041669516665622

**Published:** 2016-09-08

**Authors:** Stefano Mastandrea, John M. Kennedy

**Affiliations:** University of Roma Tre, Italy; University of Toronto, Canada

**Keywords:** vision, illusion, horizontal–vertical, size, optic slant

## Abstract

A new everyday visual size illusion is presented—the Pot/Lid illusion. Observers choose an unduly large lid for a pot. We ask whether the optic slant of the pot brim would increase its apparent size or if vision underestimates the size of tilted lids.

The pot is on the stove. The lids are in the drawer. You open the drawer, seeing disordered lids of different sizes, many set nearly vertical to help with drying. You take the one that looks correct: too big! You try again with one that looks one size too small, and—surprise!—it matches the pot exactly. A few days later you repeat the mistake. Sometimes you are right at the first go, but only if you look at the lids carefully. Sometimes your strategy is to choose one that looks a tad small, finding it is the correct one.

We call this illusion the “Pot/Lid” illusion. A “size illusion” (Robinson, 1972), it happens reliably in everyday life, like the well-known moon illusions: In one, the moon looks bigger at the horizon then at the zenith (Kaufman & Rock, 1962); in a second, because of motion induction (Duncker, 1929), the moon apparently races through clouds.

Here, we demonstrate the Pot/Lid illusion and speculate about causes.

A total of 120 participants (*F* = 111, age range 19–48; mean 22.8, SD 5.0) from the Department of Education, University of Roma Tre, volunteered for the experiment. Each had normal or corrected-to-normal vision and was naïve to the purpose of the experiment.

A cylindrical steel pot with two side handles, diameter 20 cm, 15 cm height, and 5 lids, increasing in diameter by 2 cm from 16 cm to 24 cm, with 20 cm matching the pot opening. The pot and the lids were located on a table with the pot on the left and the lids inserted in a rack on the right (Figure 1).

**Figure 1.**
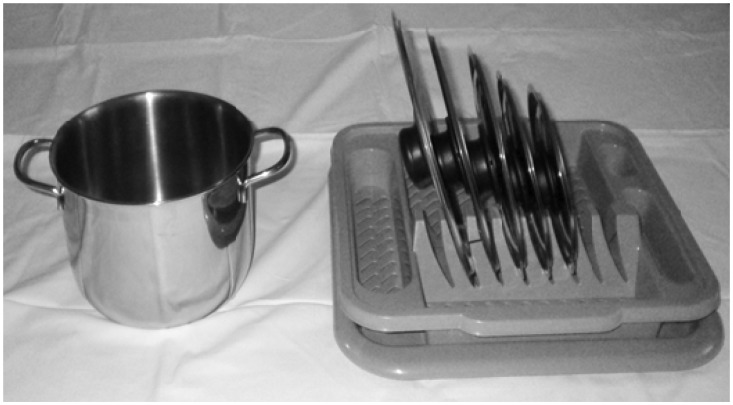
The pot and five lids (16 cm, 18 cm, 20 cm, 22 cm, 24 cm) in a rack as in the experiment.

Participants stood approximately 100 cm from the table. From this distance, the brim of the pot is a tilted and foreshortened circle. For a vantage point 60 cm above the pot brim, the brim is at an optic slant of about 60° (measured from the vertical). The set of pot-lid options from which to choose are seen end-on, inclined about 15° to the vertical.

Observers indicated which of the five lids matched the top of the pot. There were three different left–right random orders for the lids. The set of lids is unbiased, since the mean size of the lids equals the pot opening’s size.

No one chose the smallest lid. One participant (0.8%) chose the second smallest lid (18 cm); only 27 (22.5%) chose the correct lid (20 cm); 77 (64.2%) chose a bigger lid (22 cm); 15 (12.5%) chose the biggest lid (24 cm). In sum, 76.7% chose an unduly large lid. The mean (21.8; *SD* = 1.22; 95% CI = 1.55 to 1.99) was larger than true (20 cm); *t*(119) = 15.85, *p* < .001.

The chosen lid was generally unduly large. There could be several reasons. One factor in Figure 1 is that the lower contour of the lid is obscured. The upper contour is fully visible, and the radius of the lid is obvious, but observers may be unduly influenced by the visible amount of the lid’s diameter. Another factor is the contours themselves. The pot brim’s contours are easy to see, since the brim is convex. The lid’s contours narrow, offering a V shape. The V’s length would be underestimated if the termination of the V were taken to be short of the actual end. Another issue is the lids, tilted, are seen edge-on but both the azimuth and the z-dimension of the pot opening are exposed. Observers may match the lid with either of the opening’s dimensions. Perhaps the azimuth dimension of the opening is matched with the lid. However, the two are almost at right angles, and this might incur the classic horizontal–vertical T-illusion in which the crossbar appears larger than the stem. Alas, if the horizontal–vertical illusion were the key effect, a smaller lid than is true would be selected, the reverse of our finding. Further, the two extents being considered are spaced apart, which diminishes the horizontal–vertical illusion. Alternatively, vision may use the opening’s z-dimension, which is almost aligned with the near-vertical lid. Of interest, given its optic slant, the z-dimension is foreshortened by perspective (Kennedy & Juricevic, 2006). In Figure 1, the opening’s z-dimension is represented by a vertical dimension in the picture that is 58% of the pot’s horizontal extent in the picture. Did vision take the foreshortened 3D z-dimension of the pot as larger than is true, resulting in the opening’s 20 cm appearing like a lid’s 22 cm? Did crosstalk from tilt information increase apparent size (Mastandrea, Kennedy, & Wnuczko, 2014)? Visual “overconstancy” of this kind has been reported, but rarely, and only for extremes of perspective foreshortening (Juricevic & Kennedy, 2006). Rather, the standard argument about optic-slant is that we judge modestly foreshortened sizes correctly and when optic slant produces foreshortening of 80% and more, as optic slant approaches its upper limit, stretches of ground look shorter than true (Wnuczko, Singh, & Kennedy, 2016). Nevertheless, we mention the logical alternative that moderate foreshortening such as the 40% in Figure 1, given 60° optic slant, may have elongated apparent z-dimensions compared with vertical dimensions.

Finally, rectifying a lid’s size could reduce its vertical dimension. Figure 1 has horizontal and vertical dimensions, and lids modestly tilted to the vertical. At a tilt of 15°, a lid of 20 cm projects 19 cm on a vertical axis. If the vertical projection of the lid’s extent was compared by vision to the pot opening, a larger lid than is correct would be selected. This is an intriguing horizontal–(vertical projection) theory.

Like other everyday illusions, the Pot/Lid illusion is stable, as our experience suggests. Knowing it exists does not eliminate its vivid effects.
